# Overview of a Disease Outbreak and Introduction of a Step-by-Step Protocol for the Eradication of *Mycobacterium haemophilum* in a Zebrafish System

**DOI:** 10.1089/zeb.2018.1628

**Published:** 2019-01-31

**Authors:** Anita Rácz, Toni Dwyer, Shaun S. Killen

**Affiliations:** ^1^ Institute of Biodiversity, Animal Health and Comparative Medicine, College of Medical, Veterinary and Life Sciences, University of Glasgow, Glasgow, United Kingdom.; ^2^ Institute of Aquaculture, University of Stirling, Stirling, United Kingdom.

**Keywords:** disease, aquaculture, sterilization, sanitation, bacteria, zoonoses

## Abstract

In 2017, the zebrafish unit at University of Glasgow experienced a detrimental outbreak of pathogenic bacterium, *Mycobacterium haemophilum*. The presence of other bacterial species was also confirmed by bacteriology growth in the same unit. The affected individuals composed of a wild-origin parental population sourced from India and their F1 offspring generation. Bacteria were diagnostically confirmed to be present systemically in fish and within the water and biofilm of the recirculating zebrafish system. In the absence of a publicly accessible step-by-step disinfectant protocol for these difficult-to-eliminate pathogens, we devised a successful procedure to eradicate mycobacteria and *Aeromonas* species after colony removal using Cleanline Chlorine tablets (active ingredient Sodium dichloroisocyanurate) and Virkon Aquatic^®^. Postdisinfection diagnostics did not detect pathogens in the system or in the new fish inhabiting the system that were tested. Newly established fish colonies have not shown similar clinical signs or disease-induced mortality in the 1-year period following system disinfection and repopulation. We present a historical background of the bacterial outbreak and a disinfection method which can be replicated in other zebrafish facilities—at small or large scales—for reliable mycobacterium removal. This procedure can be implemented as a disinfection protocol before the introduction of a new fish population to a previously contaminated system.

## Introduction

Zebrafish (*Danio rerio*) are the second most popular animal model used in medical research after rodents.^1^ Their popularity stems from their robustness over a wide scope of water parameters and their relative ease of housekeeping and husbandry needs.^2^ Zebrafish are a shoaling species and can thus be kept in groups of up to 50 individuals per 10 L with no adverse effects on welfare.^3^ They are also an interesting species genetically, as their genetic makeup is not too dissimilar from humans.^4^ This, along with natural embryo transparency for ease of monitoring manipulations and developmental effects, has resulted in zebrafish becoming a popular species for genetic and central nervous system research.^5^

More recently, zebrafish have also been used in a range of ecological and evolutionary studies, with a growing interest in the use of wild zebrafish due to the genetic variation present within natural populations. The native range of zebrafish spans across central and southern Asia. Due to their robustness and adaptability for many environments, they can be found in a host of slow-moving rivers and reservoir habitats within their native range.^6^ Although desirable from a research perspective, wild-sourced fish can be problematic when transferred to recirculating systems within research facilities. Not only can they transmit pathogens from their respective native habitats but also they can be more susceptible to common opportunistic pathogens found ubiquitously in recirculation units.^7^

The nontuberculous mycobacteria (NTM) are generally environmental opportunistic pathogens, which are normal inhabitants of drinking water supply systems.^8^ The ability of NTM species to form biofilms and grow easily in fresh or brackish waters allows these slowly growing bacteria to persist and grow in pipes and aquarium surfaces.^9^ Modern zebrafish holding systems are designed for fish maintenance but are also an ideal habitat for opportunistic and pathogenic agents, particularly various *Mycobacterium* spp. Despite recirculating systems being equipped with mechanical, chemical, and biological filtration, inefficiencies can occur. The tropical water temperature of zebrafish units (27–29°C) provides an organically rich environment with aerobic and anaerobic compartments, which harbors a perfect breeding ground for many bacterial species.^10^

Fish are in direct contact with their aquatic environment, and thus, any external damage, combined with excess stress, can allow for quick infection of opportunistic pathogens, particularly various *Mycobacterium* spp.^11,12^ Most mycobacterial species have low virulence and commonly occur in zebrafish units without serious problems. Other species, such as *M. marinum* and *M. haemophilum*, are extremely pathogenic and, once present in a facility, can cause up to 30%–100% mortality.^11,13^ In cases when these species result in high mortality-level outbreaks, full population culling is recommended followed by thorough cleaning and disinfection of the holding system.^10^

Even after removal of all fish from a contaminated system, however, complete eradication of remaining mycobacteria (postdisinfection) within recirculating systems before introducing new fish is extremely challenging, and recurring breakouts can persist in replacement colonies.^10,14^ Mycobacteria can be highly resistant to disinfectants and cleaning products as they have a thick protective lipid layer in their cell wall.^15^ This often results in their persistence in biofilms and complex filter surfaces, even after cleaning.^11^

Mycobacterium is an acid-fast bacillus and its presence can be viewed in histological samples by staining with Ziehl–Neelsen (ZN) stain.^11^ Another clinical sign of mycobacteriosis in fish is prominent granulomatous areas in the tissues and organs, especially the kidney and liver,^16^ although diffuse systemic infections without prominent granulomas are also reported for both *Mycobacterium haemophilum* and *M. marinum.*^11^ A variety of molecular diagnostic methods have now been developed for detecting and identifying *Mycobacterium* spp.^11,16^ More recently, there are several health diagnostic companies that provide species-specific diagnostic confirmation of *Mycobacterium* spp. presence, for zebrafish health monitoring.

Identifying *Mycobacterium* spp. through routine bacteriological plate incubation and growth methods can be a challenging process. Some of the species are very slow growing (∼30-day incubation at 28°C) even when using selective agars such as Middlebrook 7H10, often used for *M. marinum* and *M. haemophilum.*^11^ To facilitate growth of mycobacteria during incubation, it is recommended that cultures are incubated at the same environmental temperatures as the aquaria from which samples were collected.^16^

While there are many recommendations online for zebrafish system disinfection postexposure to pathogens, there is currently no step-by-step procedure available. In this study we describe a detailed protocol for the eradication of mycobacteria which was used after a pathogen outbreak in a wild zebrafish colony at the University of Glasgow in 2016/2017. A high bacterial load in the fish holding units, combined with high susceptibility to the disease, resulted in the loss of almost the entire fish colony. After a few months of attempting to treat the outbreak, the decision was made that the remaining population would be culled. Before recolonizing the system with new fish to continue with the project, the facility had to be completely cleaned and the bacteria totally eradicated to prevent an outbreak from reoccurring.

Our aim is to provide a detailed disinfection procedure for the effective elimination of *M. haemophilum* and *A. hydrophila* from research-standard zebrafish units. The protocol in this study was applied for two 340 L MBKI Aquatic Habitats Z-hab stand-alone units, but this practice can be easily applied at smaller or larger scales in fish facilities in the event of mycobacterial infection. We also used the same protocol with minor modifications for the disinfection of six 300 L glass aquarium large tank units. The protocol will be useful for disinfection process in zebrafish facilities, especially for quarantine systems, where general cleanouts are required because of the frequent repopulation with newly arrived fish.

## Methods

### Animals

**FIG. 1.**
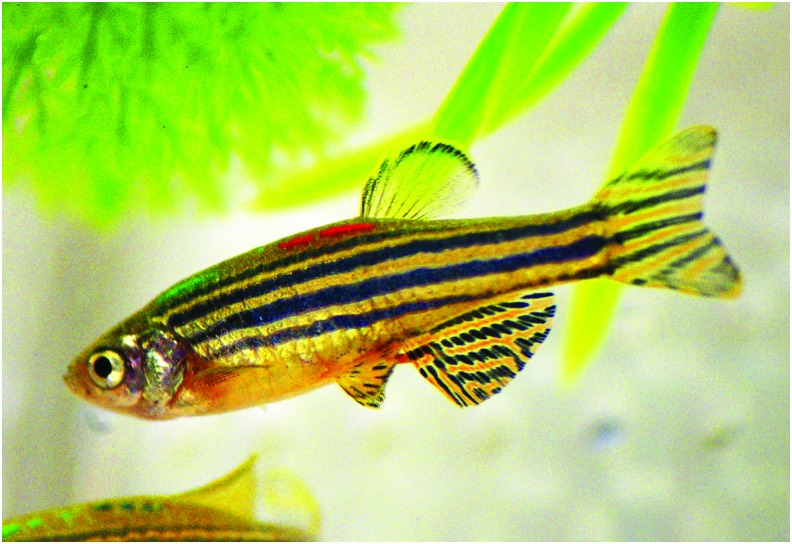
A zebrafish tagged with elastomer injected beneath the skin. Visible are two colored marks (*green* and *red*) on the dorsal side. Photo credit: A. Rácz. Color images are available online.

In 2015, a wild-origin population of zebrafish was required at the University of Glasgow to provide genetic diversity for an investigation of behavioral trait heritability, within the Institute of Biodiversity Animal Health and Comparative Medicine. Approximately 2500 wild zebrafish were sourced from the Kosi River, India. They remained in quarantine holdings for 2 months and arrived at the facility at the beginning of February 2016. A subset of the original wild parental generation was initially used for pilot studies before breeding commenced. The eggs were surface sanitized with bleach, following a standardized zebrafish egg disinfection protocol (e.g., 50–70 ppm sodium hypochlorite for 10 min^17^), before the introduction to an independent recirculation zebrafish system for rearing. The F1 offspring were produced in May/June 2016 and were reared for use in behavioral and selection experiments. The F1 generation developed well with no initial health issues arising. Holding and husbandry conditions can be seen in Table 1. The fish were to be used in behavioral and heritability studies for which they would need to be individually identified (both parental and offspring population). To make identification easier, it was decided that the fish would be tagged with four color combinations on their dorsal side using visible implant elastomer (VIE) tags from Northwest Marine Technology, Inc. (Fig. 1). This method involved placing the fish under anesthesia and injecting colored ink under the skin, which results in small wounds at the injection sites. Visible elastomer tagging is a routine identification method that is particularly common in fish studies, including small-sized fish.^18^ The F1 generation of fish was tagged in late November 2016 following a VIE tagging process where needles were sterilized with 70% ethanol between different individuals.

**Table 1.** Summary of the Water Quality Parameters and Husbandry Routine in the Facility

	*Z-hab units*	*Flow-through systems*
Water temperature	27.5–28.5°C	27.5–28.5°C
pH	7.0–7.5	6.8–7.3
Conductivity	350–400 μS	150–200 μS
NO_2_	<0.05 mg/L	<0.05 mg/L
NO_3_	<15 mg/L	<10 mg/L
Ammonia	<<0.05 mg/L	<<0.05 mg/L
Fish feeding regime >4 months	2 × daily	2 × daily
Fish feeding <4 months	4 × daily	ND
Tank cleaning regime baby-juvenile	<1 Month-daily; >1 month-weekly	ND
Tank cleaning regime adults	Monthly (change tanks)	3–4 Weekly (clean and siphon)
Adult fish holding density	3–4 fish/L	0.5–1.5 fish/L
Light cycle	13 h L:11 h D	13 h L:11 h D

ND, no data present.

### Housing and husbandry

**FIG. 2.**
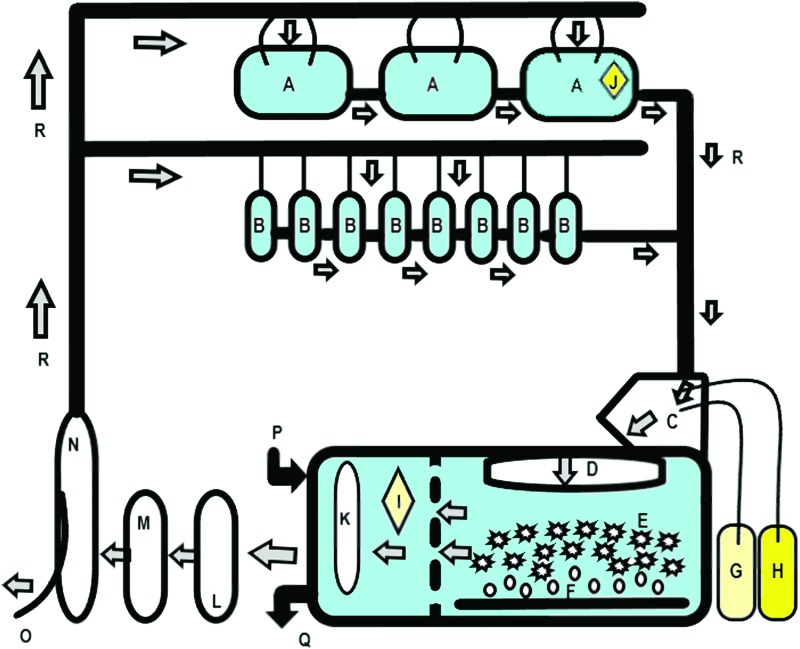
Schematic diagram of the recirculation stand-alone system design. The original Aquatic habitats Z-hab system contains five shelves to house either 6 × 10 L tanks or 12 × 3 L tanks per row. This diagram illustrates the basic features of the stand-alone unit, which includes all the important equipment such as filtering and buffering units. (A) 10 L tanks, (B) 3 L tanks, (C) spillway (backflow connector container), (D) 120 μm prefilter pad, (E) combined moving and submerged-bed biological filtration, (F) aeration from air pump, (G) conductivity dosing unit, (H) pH dosing unit, (I) conductivity electrode, (J) pH electrode, (K) heater unit, (L) 50 μm filter cartridge, (M) chemical filter (activated carbon), (N) UV disinfection lamp, (O) post-UV tap, (P) water intake valve, (Q) water exchange valve, and (R) flow direction in system. Color images are available online.

The main room and water temperature were both controlled within a range of 27°C ± 1.5°C. The light and dark cycle for the room was 13:11 h (L:D), including a 30 min transition period to simulate dawn and dusk, with a centrally-controlled separate light system on top of each row of the Z-hab units and each large holding tank. F1 generation was housed in two separate MBKI Aquatic Habitats Z-hab System zebrafish recirculation stand-alone units, with a total water volume of 340 L per unit (one rack system can house either thirty 10 L tanks or sixty 3 L tanks at a time; Fig. 2). The systems were supplied with particle and carbon filtered water with a daily exchange of 20%. All water parameters that were maintained in the facility are presented in Table 1. Conductivity and pH were monitored continuously using pH and conductivity controller units, and values were recorded daily.

A Z-hab rack system hosts a five-stage filtration unit: First stage—120 μm prefilter pad; second stage—combined moving and submerged-bed biological filtration (over 90 m^2^ of surface area); third stage—50 μm filter cartridge; fourth stage—activated carbon adsorbs volatile organics and other contaminants; and a final fifth stage as UV disinfection dose of 110 mJ/cm^2^ at the end of lamp life.

Fish under 4 months of age were reared and housed in 3 L holding tanks as a standard rearing practice in a density of 10–15 fish per liter for larvae and early juvenile stage.^19–21^ After reaching late juvenile-adult age (4 months) fish were transferred into 10 L holding tanks on the same system at a maximum density of 3–4 fish per liter. Zebrafish encounter plant life in their natural habitat and often use them as shelter, so at this point, each 10 L tank was provided with two identical (aquarium safe-preuse disinfected) plastic plants as a form of enrichment.

The wild parental generation fish were housed in six separated 300 L flow-through glass aquarium system tanks, each fitted with independent external canister filters (Hagen Fluval Fx5 External Filter) and UV sterilizers (V2ecton 600). These tanks are in the same aquaria room as the Z-hab stand-alone systems. All tanks were equipped with internal thermostat heaters at 28°C and the room temperature itself maintained at 26°C ± 1.5°C as a backup, should internal heaters fail. The larger glass aquarium systems also benefited from environmental enrichments such as (aquarium safe, disinfected) plastic plants and sand substrate (Unipack Silver Sand) to represent a more natural habitat for the wild fish.

The wild zebrafish were very timid compared to laboratory strains, and human presence often agitated them. It was decided that they would therefore be kept at a lower stocking density (max 3–4 fish per liter for the F1 generation; and 1–2 fish per liter for the wild-origin adult population) than is generally recommended for zebrafish (five fish per liter).^19–21^

In the first 4 months of life (rearing period), fish were fed four times per day, twice with differing types of dry powder baby food (NovoTom; Sera Micron; TetraMin baby, TetraMin Junior; ZM 000,100,200) and twice with 24 h freshly hatched artemia nauplii (Sanders Great Salt Lake Artemia Cysts). Late juvenile and adult fish were fed twice per day, once in the morning with differing types of flake or pellet food (TetraMin, ZM 400; ZM Granula) and once at the end of the working day with 2-day-old live freshly hatched artemia nauplii (Sanders Great Salt Lake Artemia Cysts).

For the Z-hab systems, all tanks were replaced and cleaned, as in step one of the disinfection protocol, as and when needed (generally once every 1–2 months for fish older than 3 months). Lids and baffles were cleaned more frequently as food often accumulates when feeding. Tanks containing larvae and early juvenile fish were cleaned more frequently on a daily basis for the first month and 1–2 times per week after (tanks were brushed, and dirt was collected using an automatic pipette). For the adult wild population in the flow through glass systems, cleaning occurred every 3–4 weeks (fish stayed in tanks, sides of tanks were brushed, and dirt was siphoned). Separate dedicated nets were used for Z-hab units (one for each unit) and for the adult wild fish. Temperature, pH, and conductivity were monitored daily; other water parameters such as ammonia, nitrite, and nitrate level were checked on a monthly basis. Water parameters and husbandry details can be found in Table 1.

### Pathogen outbreak

After 3–4 months of holding and testing, the parental generation in the facility began to experience higher mortality and persistent symptoms within the population such as raised scales, ulcers, ascites, and hemorrhage. After histology reports of different agents (including a possible *Mycobacterium* species presence), as a precaution the parental generation was culled from the facility. The euthanizing method used consisted of an overdose of buffered 350 mg/L concentration Tricaine (MS-222) for 30 min, followed by the confirmation of death, for example, severing the spinal cord or onset of rigor mortis. During the early stages of the disease, visually healthy individuals were bred with the aim of generating genetically diverse and unrelated families.

**FIG. 3.**
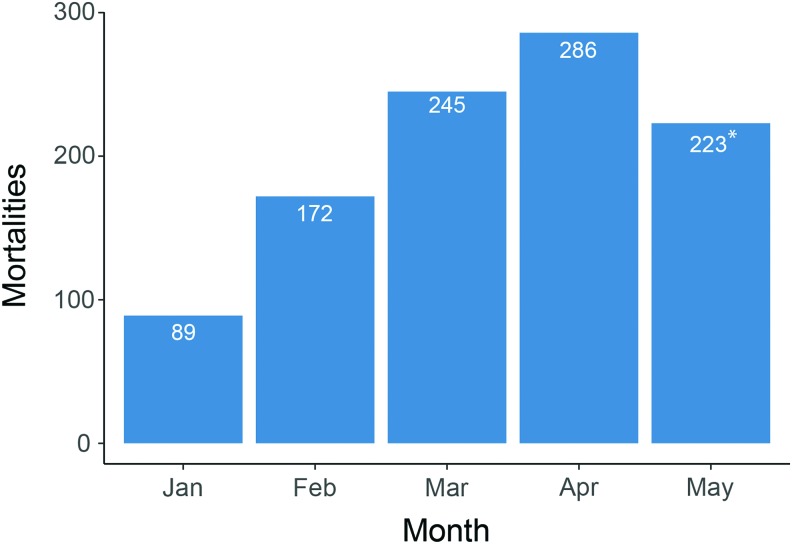
Monthly mortality of the F1 generation post-tagging in 2016/2017. Tagging occurred in late November 2016; mortalities began in December and increased successively by months. *Mortalities recorded for May 2017 represent 8 days; after this point all remaining fish were culled. Color images are available online.

Discovering a successful breeding protocol for the wild fish was challenging but after many trials, a F1 population was finally generated. Each family was created with a unique pairing of male and female crosses. The eggs were surface sanitized with bleach, following a standardized zebrafish egg disinfection protocol (50–70 ppm sodium hypochlorite for 10 min^17^). The F1 generation experienced no difficulties or health issues for the first 5 months and developed normally. The first instance of clinical signs of disease and mortalities began to occur within a few weeks post-VIE tagging, which escalated over the following months (Fig. 3). During the F1 outbreak, several egg disinfection methods were trialed, and a protocol was devised by adapting protocols described in Chang *et al*.^22^

Mason *et al.* reported an outbreak of *M. marinum* with severe detrimental effects on a zebrafish population.^23^ Armed with this information, several health and safety measures were incorporated at the University of Glasgow's aquaria facility to protect personnel and to attempt to inhibit the further spread of disease. The use of personal protective equipment such as gloves and overshoes was introduced. More frequent hand washing with soap effective against *Mycobacterium* spp. was practiced. Tank cleaning and replacing frequency were increased, net dips were instated, facility surface disinfection methods were put in place, and any individuals displaying clinical signs in the main population were immediately removed.^11,23^

### Histology and bacteriology

In the original adult wild population, clinical signs of disease began in May of 2016. After high mortalities and constant clinical signs throughout the population (e.g., raised scales, swollen abdomen, and lethargy), the on-site veterinarian made the decision to send six live individuals displaying clinical signs for histopathological examination to the Institute of Aquaculture, University of Stirling.

In the F1 population, clinical signs of disease began to occur in early 2017. An in-house investigation process ensued. Two biofilm and fish lesion swab samples were stained with ZN acid-fast stain. Results revealed acid-fast bacilli within the samples and reinforced suspicions of the presence of mycobacteria. After the in-house staining, 20 fish were selected from quarantine, all of which displayed different clinical signs of disease such as raised scales, ulcers, ascites, lethargy, emaciation, and hemorrhage.

The original F1 population consisted of 1400 fish at the sampling time with 80 individuals quarantined displaying clinical signs. From this quarantined group, 20 individuals were selected and transported to the Institute of Aquaculture, University of Stirling for histological and bacteriological investigation. Fish were sent live in an aerated container, and no deaths occurred in transport. Staff at the Institute of Aquaculture selected 6 individuals from the pool of 20 fish for histological and 3 individuals for bacteriological examination. Three inoculated trypticase soy agar or tryptone soya agar (TSA) agar plates, sampled from blood and kidney tissues of remaining zebrafish, were submitted for analysis on February 15, 2017. Two sagittal sections of the head and four transverse sections of trunk were processed for routine histopathological examination. Briefly, 5-μm-thick wax-embedded tissue sections were cut and stained either with hematoxylin and eosin or ZN stain.

### Molecular confirmation

Two F1 population zebrafish displaying clinical signs of systemic mycobacteriosis were culled with an overdose of 350 mg/L Tricaine (MS-222) for 30 min followed by confirmation of death. These fish were immediately transferred to sterile 5 mL microtubes and appropriately labelled with their elastomer identification tag and date. Two biofilm swabs were taken from each Z-hab unit. Biofilm swab sticks and instructions were provided by Charles River Laboratories. The swabs were collected from the part of the recirculation unit that displayed the largest buildup of organic matter, the spillway container (backflow effluent water collector container that collects the outflow water from the whole unit before entering back into the sump). The swab collection was concentrated at the air–water interface of the spillway container (Fig. 2).

After the histology report confirmed histological and bacteriological evidence of mycobacterial infection in the fish, a Zebrafish Mycobacterium PCR Panel molecular diagnostic test from Charles River Laboratories was requested. Charles River Laboratories provide a generic *Mycobacterium* spp. PCR Panel screening that tests each sample for the six most common species of mycobacteria: *M. chelonae*, *M. fortuitum*, *M. haemophilum*, *M. marinum*, *M. peregrinum*, and *M. abscessus*. The company uses the highly sensitive TaqMan^®^ RT-PCR method to detect genetic material from contaminated agents, greatly reducing the number of false positives. Appropriate positive and negative controls were used as per company protocols. For sample preparation, the company ground and homogenized the whole body of the fish in buffer and used this for total nucleic acid purification.

### System disinfection protocol

For all portions of the protocol, appropriate personal protective equipment was worn, particularly when working with chlorine bleach. This included protective covering for skin, eyes, face, clothing, in the form of long-sleeve gloves, face masks, and goggles). Additional equipment for the protocol included Cleanline chlorine tablets (Prime Source) and cleaning materials (sponges and pipe/bottle brushes). The specific steps of the disinfection protocol are as follows:

(1) Soak all tanks, baffles, and lids in bleach (minimum concentration 200–250 ppm) for 2 h before scrubbing and rinsing with hot water (∼50°C). All parts should be air-dried before reuse. More than one set of tanks and materials for each unit are usually present in research facilities. Therefore, it is advised to clean and dry all tanks thoroughly so that they can be removed and stored elsewhere before commencing room disinfection. Please note that many companies supply zebrafish recirculation units and have manufactured tanks that can be cleaned in cage washers. Some companies design tanks which can also be autoclaved but due to their large size (3 L; 10 L) this is often not feasible, especially in smaller facilities. It is also good practice to clean contaminated systems such as quarantine units separately, to avoid contamination to the main facility; therefore, manual tank cleaning should be involved in the disinfection process.
(2) Day 1—set system water exchange to 0%, switch off air pump and UV filtration, and remove activated carbon and filter materials. Turn off water pump before emptying all water from the system. Empty the tanks manually. Remove filtration materials (e.g., Siporax panels and static media) and autoclave before disposal.
(3) Fill system with tap water. Add chlorine tablets (e.g., Cleanline Chlorine tablets from Prime Source—active ingredient Sodium dichloroisocyanurate) to the sump for a concentration of 250–300 ppm. Consider the concentration for the total system volume and not just the sump alone as this will dilute the concentration, perhaps below effective levels. Follow manufacturer guidelines for the number of tablets required for the total volume of system water. Wear face mask to avoid inhalation of chlorine gas. Dissolve tablets completely before turning on the system pump. Do not use air stones to dissolve the tablets; this will release more gas into the room. Keep all lids and coverings in place across the whole system to avoid chlorine gas evaporation and aerate the room as much as possible.
(4) Run system with dissolved tablets for 1 h. Turn off water pump and leave motionless for an additional 1 h. Repeat this full process 1 × more. Add 5%–10% extra tablets (to compensate for previous evaporation) and run system for another hour before turning off and leaving idle overnight with the chlorine in the system.
(5) Day 2—empty system completely. Be aware of splash backs and take extra precautions by wearing appropriate protection for skin, eyes, clothes, and face. Repeat steps 3 and 4.
(6) Day 3—wearing appropriate protection, empty tanks, and store them until system scrubbing is complete. Prepare 200 ppm (minimum) bleach solution and scrub all reachable system parts, using pipe/bottle cleaners for pipes. Empty sump and scrub every surface with brushes and sponges. Put tanks back in place. Fill system completely and run with copper free tap or filtered water for 2–3 h. Empty out then fill again with fresh water (water type optional but must be copper free). Set water exchange on system to 80% and leave to run overnight.
(7) Day 4—set water exchange back to 0%. Repeat the empty and refill process with water. Add Virkon Aquatic^®^ to the system to a concentration of 0.1–0.2 g/L and start water pump. After 2–3 h of running, empty system and refill with fresh water (water type optional but must be copper free). Set water exchange to 80% and leave running overnight.
(8) Day 5—empty system and wash/scrub surfaces and pipes with hot water (∼50°C). Fill system with RO- reverse osmosis (purified water) or particle and carbon filtered water, add prefilter units (filter pad and 50 μm filter), and run for 2–3 h. Empty system then refill with RO or particle and carbon filtered water. These actions should take a full day, so at the end of the work day, add new activated carbon and run system at 80% water exchange for 2 full days.
(9) Day 8—empty system completely and remove tanks. Clean tanks as per step 1. If you have replacement/spare sets of dry clean tanks, install these on the system. Alternatively, install the previously cleaned and dried tanks. Discard all prefilters and activated carbon and replace with new stocks. Leave system to air-dry completely (∼5–7 days).
(10) Once completely dry, fill with particle and carbon filtered water (or RO) and add new static media and Siporax to the sump tank. Turn on air pump and allow static media to soak for 1–2 days. Run the system on 50% water exchange for the first few days. Test the system water with a standard Chlorine test (e.g., Hanna Instruments; kit H1-3831F) for traces of remaining chlorine. This should be carried out daily for a week, collecting water from different locations of the system. Run system as normal with the UV sterilizer switched off. If no trace of chlorine is detected, revert the water exchange to the original 10%–20%. Begin buffering the system to the desired pH and conductivity levels.

It is common in newly primed systems for ammonia and nitrite peaks to occur if biological filtration is not active or not working sufficiently; to avoid this situation take the following action. Add start-up nitrifying bacteria to biofilter unit and run system for 3–5 days without UV to allow bacterial communities to colonize (Prodibio-BioDigest or ATM Colony Nitrifying Bacteria). The bio-filtration bacteria have successfully colonized the system and are considered to be working when the prefilter pad begins to show signs of color. At this point, introduce either larval fishes or a small number of adult fish to the system.

For the introduction of larval fish, normal introduction methods and feeding regime can be used. However, adult fish should be fed sparingly (half the quantity of a normal feeding regime) for the first week to control ammonia levels, before gradually increasing both introduced fish numbers and the quantity of food. The biofilter status can be monitored with standard water quality tests to check ammonia, nitrite, and nitrate levels (such as JBL/API/Sera). This should be carried out daily until desired levels are reached.

Dosing the system weekly with nitrifying bacteria until water parameters stabilize (low level of nitrite, trace of nitrate) will ensure sufficient biological filtration. If the biological filtration is working properly, the elements of a complete nitrogen cycle will be in place, and this can be easily verified by testing for the above three nitrogen compound parameters. If no trace of nitrate is detected with increased ammonia and/or nitrite levels, then the bacterial activity of the biological filtration is still not adequate (see “Results” section).

Following the complete disinfection protocol, standard chlorine tests (Hanna Instruments; kit H1-3831F) were used to check for traces of remaining chlorine. This was carried out daily for a week, and samples were collected from multiple locations on the system (e.g., sump, UV outflow tap, different tanks, and individual tank taps). Test kit sensitivity was also tested by comparing tap water and filtered water when testing our system water. As different systems were used for fish of different ages, fish were introduced to the restarted systems in differing ways. Z-hab recirculating units were used for the rearing of larvae fish from a new population from a new colony.

For the introduction of adult fish (predisinfection, in-house, adult wild population), 300 L glass aquarium units were used. In two of these tanks, neither cautious introduction nor decreased feeding steps were implemented. Approximately 500–600 fish were simultaneously introduced per tank, and nitrifying bacteria displayed signs of inefficiency (see “Results” section). To prevent biological filter issues from arising when introducing a high number of fish at once for the remaining experimental group, these fish were split into four 300 L glass aquarium units, and a new introduction protocol was followed: A maximum of 100 fish were added to each tank every other day while implementing a half-ration feeding regime for the first week.

### Postdisinfection diagnostics

The QM Diagnostics Circulum Kit included test tubes for the following: one biofilm swab, one tube for a water sample, one fish sample, and one tube for a food sample. In November of 2017, 6 months after the sterilization of the facility, one full testing kit was filled (separately for each of the stand-alone and recirculation unit), as instructed by the company and returned for full diagnostic investigation. Food samples included both live and dry food sample types. One test kit contained freshly hatched artemia nauplii and in the other kit dry food (ZM400) for testing.

The biofilm swabs were collected from the part of the recirculation unit that displays the largest buildup of organic matter, the spillway container (Fig. 2). Two fish were randomly selected from the system. The fish were culled according to United Kingdom Home Office Schedule 1 guidelines for humane euthanasia (overdose of 350 mg/L buffered Tricaine solution (MS-222) for 30 min, followed by confirmation of death). The fish were stored and processed by QM Diagnostics as per company diagnostic guidelines. Circulum sampling profile consists of high sensitivity, highly specific, and fast TaqMan qPCR and chemical assays, which are all validated. In the sample preparation process, the company grinds and homogenizes the whole body of fish in buffer and uses this for total nucleic acid purification.

Following positive *Mycobacterium* spp. detection in the standard kit profile, a decision was made to perform species-specific testing for *Mycobacterium* spp. screening, to detect the possible presence of the six most common species: *M. chelonae*, *M. fortuitum*, *M. haemophilum*, *M. marinum*, *M. peregrinum*, and *M. abscessus*.

In June of 2018, a second round of diagnostic tests was carried out on both systems. This was ∼1 year after carrying out the disinfection protocol and 6 months from the first postdisinfection diagnostic test. Following the recommendations of two detailed methodology articles providing newly established health monitoring techniques in zebrafish facilities, we used these environmental screening techniques for our samples.^24,25^

A total of five biofilm swabs were taken from both Z-hab units. A swab from each unit was taken from the spillway container and directly from the biofilm in the sump tank (from a 10 to 20 cm area between the water–air surface layer in the mid section of the sump). The fifth swab was taken from the inner-end section of the backflow pipe from one Z-hab unit (Fig. 2), the unit which housed the majority of the reared fish. Each time the biofilm swab was taken, the collection area was concentrated at the air–water interface of the sump tank.^25^

Five rearing tanks from the units housing the new population were selected randomly for fish sludge collection. The smaller sized baffle mesh (400–700 μm) used in rearing tanks accumulates sludge more readily, resulting in a concentrated layer at the bottom of each tank. This made for straightforward collection, and samples were stored in 15 mL tubes. Each sludge sample swab was then prepared for PCR following routine protocols.^24,25^ All 10 samples (five biofilm swab and five sludge swab) were sent to QM Diagnostics for PCR testing. Samples were sent to the company in separate tubes and then pooled by the company upon receipt. Pooled samples were based on an in-house validated pooling protocol by QM Diagnostics where five individual samples were pooled into one test sample for each category (biofilm or sludge).

For PCR, all samples were pooled in sterile phosphate-buffered saline buffer, and total nucleic acid was extracted. Swabs were suspended and mixed and then 175 μL of the pooled sample diluent was used for the DNA extraction. As mentioned previously, the PCR profile consists of high sensitivity, highly specific, and fast TaqMan qPCR and chemical assays, which are all validated by QM Diagnostics Company standards.

## Results

### Histology

Infected individuals in the original wild parental population showed extensive granulomatous inflammation forming sheets of macrophages within the dermis, musculature, liver, and peritoneum and extending to all visceral organs. In the majority of fish, distinct granuloma formation was also noted. Macrophages generally had a finely granular eosinophilic to basophilic cytoplasm, suggestive of bacterial infection. Large numbers of intralesional acid-fast, predominantly Gram-positive bacteria were seen within sheets of macrophages and within distinct granulomas.

Histological examination of F1fish revealed copious basophilic, granular bacterial colonies within the epidermis, muscle tissues, liver, and peritoneum and extending to all visceral organs. Often associated with the granuloma formations were extensive inflammations. In five of the six samples, ZN stains were strongly positive for mycobacteria within the lesions. A lack of staining and bacteria identification within some lesions occurred, but this was thought to be artifact rather than a secondary bacterial infection. It was advised by histology staff that a severe systemic mycobacterial infection can often be associated with epidemics and high mortalities if the disease pressure is sufficient and/or there are underlying stressors. It was also noted that the weight loss and mortalities seen in the source populations were likely related to this.

### Bacteriology

Bacteriology resulted in mixed growth on all samples. Subculture of the dominant colony type from all three fish samples was identified as a Gram negative, short rod, oxidase positive, fermentative motile bacterial species with a biochemical profile matching motile *Aeromonas* species, likely *A. hydrophila*. None of the samples was positive for recovery and identification of *Mycobacterium* spp. using conventional tests.

### System disinfection protocol

From chlorine testing, no trace of chlorine was detected at any point, even during initial sampling. Test kit sensitivity results showed varying amounts of detectable traces of chlorine between water sources. As previously described, the introduction of adult fish into the first two tanks did not involve either cautious introduction or decreased initial. After this introduction, ammonia, nitrite, and nitrate remained minimal for several days, then ammonia peaked for 2–3 days (0.4–0.6 mg/L), but after 50% water change returned to undetectable levels. This was quickly followed by a nitrite peak (0.8–1 mg/L) with undetectable nitrate levels.

This demonstrated that the nitrifying bacteria were beginning to colonize the system, but the number of bacteria was not yet sufficient enough to manage the high load of nitrogen waste being produced by the fish. After another 50% of water change and the addition of additional nitrifying bacteria, nitrite levels started to decrease and in a weak time stabilized below 0.1 mg/L; nitrate levels reached and stabilized at 1–5 mg/L. After using the new introduction protocol of splitting the experimental fish into four 300 L tanks and introducing a maximum of 100 individuals per every second day with a half-ration feeding regime, there were no high peaks of ammonia detected, and the detectable level of nitrogen compounds was low. Any increases were normal and constant until they reach stable levels as shown in Table 1.

### Molecular diagnostics

Predisinfection, PCR testing confirmed *M. haemophilum* presence in both the tested biofilm and frozen fish samples (Table 2). *M. chelonae* was present in the biofilm sample, but not in the fish sample, and all tested samples were negative for all the other *Mycobacterium* species.

**Table 2.** Summary of Bacterial Presence in the Zebrafish Unit, Before and Six Months After the Described Disinfection Protocol

*Species*	*Biofilm*		*Water*		*Fish*	
	*Before*	*After*	*Before*	*After*	*Before*	*After*
*Mycobacterium haemophilum*	2/2	0/2	ND	0/2	2/2	0/2
*Mycobacterium chelonae*	2/2	2/2	ND	0/2	0/2	0/2
*Aeromonas hydrophila*	ND	0/2	ND	0/2	ND	0/2

After a mycobacterium-positive histopathology report on fish samples, a customized polymerase chain reaction (PCR) panel was performed to identify species. Postdisinfection, a circulum sampling PCR panel was used for full system diagnostics. *A. hydrophila* presence in the system and fish was confirmed by histology and bacteriology test, but was not included in the predisinfection PCR panel test, only in the postdisinfection testing.

Zero (0) represents a negative result for the tested pathogen.

ND, no data were present on PCR test (not tested by PCR).

Postdisinfection (after 6 months) PCR results confirmed that artemia (live food sample), water, and fish samples were negative for *A. hydrophila* and all *Mycobacterium* species tested, including *M. haemophilum*. Dry food sample (ZM400), however, tested positive for *A. hydrophila*. In more detail, both Z-hab systems, water, and fish samples were negative for all the tested *Mycobacterium* species together with all the other tested species, including *A. hydrophila*, *Flavobacterium columnare*, *Pseudocapillaria tomentosa*, *Pseudoloma neurophilia*, *and Pseudomonas aeruginosa*. The biofilm samples alone tested positive for *M. chelonae* and were negative for all other tested species.

Second postdisinfection (after 1 year) PCR results confirmed that *A. hydrophila* and *M. haemophilum* were not present in all the tested samples. For this test, pooled environmental samples were used to cover a wider range of testing sites and areas of the systems. The test was tailored to specifically test just for *A. hydrophila*, *M. chelonae*, and *M. haemophilum*. *A. hydrophila* and *M. haemophilum* were not present in any of the tested environmental samples. *M. chelonae*, however, was present in the tested environmental samples, as it was 6 months previously. The pool of biofilm and sludge both tested positive for *M. chelonae* and negative for *A. hydrophila* and *M. haemophilum* (Table 3).

**Table 3.** Results of Bacterial Testing in the Zebrafish Unit One Year After the Disinfectant Protocol Was Implemented (Half a Year After First Molecular Diagnostics Test)

*Species*	*Biofilm*	*Sludge*
*M. haemophilum*	Not present	Not present
*M. chelonae*	Present	Present
*A. hydrophila*	Not present	Not present

For each type of sample (biofilm or sludge), five individual samples were collected (5 × biofilm, 5 × sludge, 10 samples in total). The samples were pooled into a combined test sample by the diagnostics company (combining the five biofilm samples for one test and the five sludge tests as another) for PCR. As this was a pooled test of all five samples, pathogens are deemed as being either present or not present.

## Discussion

The disinfection protocol described in this study was successful for system eradication of the pathogens *M. haemophilum* and *A. hydrophila*. A previous disinfection study by Whipps *et al.*^11^ used 1000 ppm bleach (buffered to pH 7) to sanitize a contaminated aquatic housing system after an outbreak of *M. haemophilum*. Postdisinfection diagnostics in that study also revealed that the pathogen was no longer present, but this study lacked step-by-step details of the full disinfection process (such as contact time with bleach and method of cleaning). In another study, useful information was reported regarding the efficacy of bleach against *M. marinum* after trialing different contact times and concentrations.^14^ It was demonstrated that sodium hypochlorite, even at concentrations as low as 200 ppm, was effective to stop *M. marinum* growth within a 60-min contact time.^14^

Using this information and knowledge of other system sanitizing examples, we devised a protocol implementing an active chlorine concentration of 250–300 ppm over a prolonged contact time, repeating the process several times with and without water movement through the system. The use of Cleanline Chlorine tablets proved advantageous as they effervesce with an active ingredient of Sodium dichloroisocyanurate (with a pH of diluted solution/1 L—5.0 to 6.0) as an alternative for sodium hypochlorite.^26^ When dissolved in water, these tablets provide a measured dose of available chlorine, which can be used for the disinfection of equipment, work surfaces, and holding systems. Sodium dichloroisocyanurate has a high and rapid activity against a wide range of bacteria, fungi, spores, and viruses.^26,27^

Cleanline Tablets also offer economic advantages in terms of ease of use, accurate dilution, and prolonged shelf life. In relation of cost-effectiveness for a stand-alone unit, which is 300–350 L total volume, it is necessary to use approximately half of a bottle of tablets (around 70–80 tablets per unit). The efficacy of Cleanline Chlorine tablets alone had not been tested before the use of Virkon Aquatic, but previous work has shown that Virkon^®^ alone is ineffective for the elimination of certain *Mycobacterium* spp.^14,28^ It was possible that the original wild-origin population carried additional undetected pathogens, including other bacterial or viral species, so it was decided that Virkon Aquatic would be used as a secondary disinfectant agent in our disinfection method. This product was chosen due to its high range of antibacterial and antiviral effects, and there are a range of general recommendations for its use in aquaculture as a safe and harmless treatment for fish.^29^

Following the histopathology reports which indicated severe systemic mycobacterial infection, the presence of *Aeromonas* species was thought to be a secondary infection caused by the mycobacteria suppressing the immune system of the fish. Therefore, our attention focused on the eradication of primary mycobacterial presence rather than that of *Aeromonas* species. The presence of *Aeromonas* species, however, demonstrated the likelihood of multisecondary infection agents being present in the system, as well as the high load of mycobacteria. In the second postdiagnostic test, the 10 samples taken were environmental samples; as it has been shown previously in other studies there is higher potential to detect *Mycobacterium* spp. and *A. hydrophila* from such samples compared to that of fish tissues.^24,25^

All collected environmental samples in the systems were negative for both *M. haemophilum* and *A. hydrophila* after 12 months, and a new fish colony was introduced to the sanitized systems with no adverse health throughout this time period. The new population of fish was semidomestic adult zebrafish that arrived and was introduced to the facility from a fish farm in Singapore, at the end of May 2017. At the time of arrival, the new adult population was quarantined and held in another aquarium within the animal facilities (located in the same building) for the first few months. Once settled, the fish were bred following standard zebrafish breeding guidelines (couple crosses), producing a new F1 generation of fish.^2,21^ During the breeding process, egg disinfection was carried out using a modification of protocols described by Chang *et al.*^22^

Once the egg surface disinfection had taken place, larvae were introduced into the previously disinfected Z-hab recirculating systems. Larvae fish developed normally without any morphological or developmental problems, with a survival rate of 85%–90% at 4 months. Upon reaching 5 months of age (December 2017), 1800 zebrafish (F1 generation) were VIE tagged (implementing a newly improved protocol; article in preparation) for identification purposes. Since then, there have been no losses or evidence of disease to date. All tagged individuals are now 12 months old and are being used in continuous experimental investigations (moderate severity level) without displaying any clinical signs of disease.

The adult parental populations were also used in behavioral and selection studies and, thus, were also VIE tagged with the same newly improved protocol. These fish were then transferred to the 300 L glass aquarium systems which previously housed the wild parental population before the disinfection and repopulation. The same disinfection protocol that was used on the Z-hab stand-alone units was also carried out on the glass system units. Postdisinfection, the newly tagged individuals were slowly introduced to the glass systems, increasing the numbers every other day, as tagging progressed. During this time of reestablishment, the biofilter progress was regularly checked as in step 10 of the disinfection protocol. For the following 7 months after tagging and transferring to the glass aquarium systems, there have been no unexpected losses or clinical signs, as were seen during the disease outbreak.

From an amalgamation of our investigation results and postdisinfection observations in differing generations, we can conclude that the system sanitizing protocol we describe in this study is sufficient to eradicate both *M. haemophilum* and *A. hydrophila* from this type of facility. Postdisinfection, newly stocked fish continued to be healthy for at least 1 year. Additional evidence for the absence of these species was provided by negative results in postdisinfection diagnostic tests for bacterial presence.

We believe that the above information, and the presented disinfection protocol, will be useful for other zebrafish research facilities encountering similar issues. The protocol is easy to follow-up, and the costs of the chemicals used are quite low. Therefore, it is a cost and time effective method to use for facility disinfection before the introduction of new colonies of fish. Generally, quarantine units in fish research facilities are small- or mid-range sized units where frequent clear outs and repopulation are constantly occurring. The presented protocol in this study will be useful and beneficial, especially for the disinfection of these quarantine units, in every zebrafish research facility.

The simplicity of this disinfection protocol allows for simple adjustment to be used in different settings, such as flow-through or recirculation systems (both glass and polycarbonate designs), and can be adapted to cater to smaller or larger scales for other aquatic facilities as well. However, it is important to note that any product containing an active oxidizing agent can be slightly corrosive and, if used frequently, may adversely affect components of a fish holding system (e.g., metal fittings). Before the use of any disinfecting protocols it is always wise to consider such issues and consult with system manufacturers for advice.
